# Knockdown of MALAT1 Inhibits the Progression of Chronic Periodontitis via Targeting miR-769-5p/HIF3A Axis

**DOI:** 10.1155/2021/8899863

**Published:** 2021-02-01

**Authors:** Qinchao Chen, Meng Cao, Hanyi Ge

**Affiliations:** Department of Stomatology, Zibo Central Hospital, No. 54, Gongqingtuan Road, Zhangdian District, Zibo City Shandong Province 255000, China

## Abstract

**Purpose:**

Chronic periodontitis (CP) is a long-lasting inflammatory disease that seriously affects oral health. This study is aimed at investigating the regulatory mechanism of metastasis-associated lung adenocarcinoma transcript 1 (MALAT1) in CP.

**Methods:**

Primary human periodontal ligament cells (PDLCs) were treated with *P. gingivalis* lipopolysaccharide (LPS) to establish a CP model. Quantitative real-time PCR (qRT-PCR) was used to measure the expression of MALAT1 and miR-769-5p in gingival tissues of patients with CP and LPS-treated PDLCs. Cell viability was detected by 3-(4,5-dimethyl-2-thiazolyl)-2,5-diphenyl-2-H-tetrazolium bromide (MTT) assay. Enzyme-linked immunosorbent assay (ELISA) was used to measure the levels of inflammatory cytokines. The protein levels of caspase-3, Bax, Bcl-2, and hypoxia-inducible factor (HIF) 3A were determined by western blot assay. Dual-luciferase reporter (DLR) assay was applied to validate the target relationships between miR-769-5p and MALAT1/HIF3A.

**Results:**

The expression of MALAT1 and HIF3A was enhanced, and the expression of miR-769-5p was reduced in gingival tissues of patients with CP and LPS-treated PDLCs. MALAT1 knockdown promoted cell viability and inhibited inflammation and cell apoptosis in LPS-treated PDLCs. MALAT1 targeted miR-769-5p and negatively regulated miR-769-5p expression. miR-769-5p overexpression promoted cell viability and inhibited inflammation and cell apoptosis in LPS-treated PDLCs. Besides, miR-769-5p targeted HIF3A and negatively modulated HIF3A expression. Both miR-769-5p inhibition and HIF3A overexpression reversed the inhibitory effects of MALAT1 silencing on LPS-induced PDLC injury in vitro.

**Conclusion:**

MALAT1 knockdown attenuated LPS-induced PDLC injury via regulating the miR-769-5p/HIF3A axis, which may supply a new target for CP treatment.

## 1. Introduction

Periodontitis is a common inflammatory disease, which is caused by the imbalance of periodontal microbiota, such as *Porphyromonas gingivalis* (*P. gingivalis*) [1]. Chronic periodontitis (CP) is a long-lasting periodontitis disease and a chronic noncommunicable disease that destroys the integrity of the periodontium and leads to gingival swelling and bleeding, bone loss, and tooth exfoliation [2]. According to data from the World Health Organization, CP is one of the chronic noncommunicable diseases that seriously affect people's quality of life [3, 4]. The current treatment methods of periodontitis including scaling, surgery, and systemic antibiotics have made great progress, but the treatment effect remains dissatisfying [5]. Thus, it is necessary to understand the molecular mechanism of CP to improve its therapy.

Long noncoding RNAs (lncRNAs) above 200 nt have been recognized to be involved in many human diseases [6]. Recently, many researches showed that lncRNAs are aberrantly expressed in periodontitis and therefore play essential roles in the development of periodontitis [7, 8]. LINC00687 expression is upregulated, whereas the expression of LBX2-AS1 and LINC01566 is downregulated in periodontitis samples [9]. lncRNA PTCSC3 expression is decreased in periodontal ligament stem cells (PDLSCs) of CP patients, and its overexpression inhibits PDLSC proliferation [10]. Notably, metastasis-associated lung adenocarcinoma transcript 1 (MALAT1) is also related to periodontitis progression [11]. MALAT1 expression is obviously increased in PDLSCs isolated from periodontitis patients, and its overexpression promotes cell proliferation [12]. MALAT1 is highly expressed in inflammatory gingival tissues of CP and promotes inflammatory cytokine secretion in human gingival fibroblast (HGF) cells [13]. However, the detailed molecular mechanism of MALAT1 in CP needs further study.

MicroRNAs (miRNAs) are a type of noncoding RNAs that are implicated in some pathogenic events, including periodontitis [14]. Previous studies have revealed that some miRNAs affect the occurrence and development of periodontitis, such as miR-21 [15], miR-182 [16], and miR-155-5p [17]. Additionally, miR-769-5p acts as a molecular biomarker and is involved in some immunological disorders. Chen et al. have discovered that miR-769-5p expression is upregulated in rheumatoid arthritis (RA) and gouty arthritis (GA) [18]. Besides, miR-769-5p expression is decreased in lipopolysaccharide- (LPS-) treated periodontal ligament cells (PDLCs) [19]. Previous studies suggested that the roles of miRNAs in periodontitis are regulated by lncRNAs. For example, lncRNA TUG1 mitigates cell injury and cytokine production by regulating miR-498 in LPS-treated PDLCs [20]. The inhibition effects of miR-20a on secretion of inflammatory cytokines are regulated by MALAT1 in LPS-treated HGF cells [13]. However, the exact role of miR-769-5p and the interaction with MALAT1 in CP are unrevealed.

Generally, hypoxia-inducible factor (HIF) 3A is known as an oncogene in several types of human cancers, such as in ovarian [21], prostate [22], breast [23], pancreatic [24], and non-small-cell lung [25] cancers. In addition, two members (HIF1A and HIF2A) of the HIF family are confirmed to upregulate in aging gingival tissues with hypoxic stress [26]. Notably, a recent study conducted by Jia et al. has demonstrated that HIF3A expression is increased in CP tissues and LPS-induced PDLCs and HIF3A overexpression partly reversed the effects of miR-210 upregulation on cell viability, apoptosis, and inflammation factor expression in LPS-treated PDLCs [27]. Nevertheless, whether HIF3A interacts with the MALAT1/miR-769-5p axis in participation of CP progression is relatively unknown.

In our study, the expression of MALAT1, miR-769-5p, and HIF3A was measured in gingival tissues of patients with CP and LPS-treated PDLCs. Then, we investigated the effects of MALAT1 knockdown or miR-769-5p overexpression on cell viability, inflammation, and cell apoptosis in LPS-treated PDLCs. Furthermore, we further explored the regulatory mechanism of MALAT1/miR-769-5p/HIF3A axis on LPS-induced PDLC injury. This study may offer an underlying target for improving treatment strategy of CP.

## 2. Material and Methods

### 2.1. Gingival Sample Collection

A total of 26 patients (12 males, 14 females, median age: 36 ± 11 years old) with CP and 17 healthy controls (8 males, 9 females, median age: 32 ± 8 years old) from March 2018 to September 2019 were chosen in our hospital. The gingival tissue samples from CP patients were obtained during surgical therapy [28], and healthy gingival tissue samples from healthy controls were collected during crown-lengthening procedures. This study obtained the ratification of the local ethics committee, and written informed consents were acquired from all individuals.

### 2.2. Cell Culture and Treatment

In line with previously described methods, PDLCs were isolated from healthy periodontal ligament in the middle third of the periodontal ligament root of the third molars of 5 healthy volunteers [29]. The cells were cultured in Dulbecco's Modification of Eagle's Medium (DMEM) (Gibco, Carlsbad, CA, USA) containing 10% fetal bovine serum (FBS, Gibco), 100 U/mL penicillin, and 100 *μ*g/mL streptomycin at 37°C in an incubator with 5% CO_2_. PDLCs in the third generation were used in the next experiments. To establish a CP model, PDLCs were treated with 100 ng/mL *P. gingivalis* LPS (Sigma, St Louis, MI, USA) for 72 h.

### 2.3. Cell Transfection

Small interfering RNA negative control (si-NC), si-MALAT1, miRNA negative control (miR-NC), miR-769-5p mimics, miR-769-5p inhibitor, and pcDNA-HIF3A were acquired from GenePharma (Shanghai, China). PDLCs were planted into 6-well plates and grew to 80% confluence. The above factors were transfected into PDLCs utilizing Lipofectamine 3000 (Invitrogen, Carlsbad, CA, USA) in accordance with the manufacturer's instructions. The cells were collected at 48 h after transfection.

### 2.4. Quantitative Real-Time Polymerase Chain Reaction (qRT-PCR)

Total RNA from gingival tissues and PDLCs was obtained by TRIzol (Invitrogen), and cDNA was produced by utilizing the PrimeScript RT Master Mix (for gene amplification, Takara) or Mir-X miRNA First-Strand Synthesis Kit (for miRNA amplification, Takara). The qRT-PCR was performed by a SYBR Green PCR Kit (Takara). Primer sequences are enumerated in Table 1. The qRT-PCR conditions were as follows: 94°C for 10 min, followed by 40 cycles at 94°C for 15 s, 60°C for 1 min, and 72°C for 1 min. The expression of MALAT1 and HIF3A was normalized by GAPDH. U6 acted as the endogenous control for miR-769-5p. Relative gene expression was measured by 2^−*ΔΔ*Ct^ method.

### 2.5. Western Blot

Total proteins were extracted from PDLCs using RIPA buffer (Beyotime, Shanghai, China). Equal protein samples were separated by 10% sodium dodecyl sulfate polyacrylamide gel electrophoresis (SDS-PAGE) and then transferred to PVDF membranes (Bio-Rad, Inc., Hercules, CA, USA). Then, the membranes were blocked with 5% nonfat milk for 1 h and incubated overnight at 4°C with primary antibodies of Bax (1 : 1,000, ab32503, Abcam, Cambridge, UK), Bcl-2 (1 : 1,000, ab32124, Abcam), HIF3A (1 : 1,000, ab10134, Abcam), caspase-3 (1 : 1,000, ab32351, Abcam), and *β*-actin (1 : 1,000, ab5694, Abcam). After the membranes were washed with tris-buffered-saline Tween (TBST), a secondary antibody (1 : 5,000, ab6728, Abcam) was added to incubate with the membranes at 37°C for 2 h. The immunoblots were quantified by using ImageLab software (Bio-Rad, Inc., Hercules, CA, USA). The relative protein levels of Bax, Bcl-2, HIF3A, and caspase-3 were normalized by *β*-actin.

### 2.6. MTT Assay

Following transfection and LPS treatment, PDLCs were planted into 96-well plates (3 × 10^4^ cells/well). The MTT (5 mg/mL, Sigma) was added into each well for 4 h; then, DMSO (200 *μ*L, Sigma) solution was subjoined to dissolve formazan crystal. Finally, the absorbance at 570 nm was detected by a microplate reader (Molecular Devices; Hercules, CA, USA).

### 2.7. Enzyme-Linked Immunosorbent Assay (ELISA)

The supernatants of PDLCs with transfection and LPS treatment were gathered. ELISA kits (Boster, Wuhan, China) were used to measure the levels of interleukin- (IL-) 1*β*, IL-6, and tumor necrosis factor- (TNF-) *α* in supernatants according to the manufacturer's protocol. The absorbance at 450 nm was read using a microplate reader (Molecular Devices).

### 2.8. Target Prediction

The miRNA targets of MALAT1 were predicted using StarBase software (http://starbase.sysu.edu.cn/), and 357 miRNA targets were predicted. Among these miRNA targets, miR-769-5p was selected due to its important role in LPS-induced periodontal ligament cells [19]. In addition, the regulatory relationship between MALAT1 and miR-769-5p has not been studied yet. The mRNA targets of miR-769-5p were predicted using StarBase software and TargetScan software (http://www.targetscan.org/vert_72/). A total of 2,321 and 3,669 targets were predicted, respectively. Afterwards, HIF3A was chosen due to its crucial role in periodontitis [27] and unknown relationship with miR-769-5p.

### 2.9. Dual-Luciferase Reporter (DLR) Assay

The fragments of MALAT1/HIF3A containing the wild-type or mutant binding sites of miR-769-5p were cloned into the pGL3 vector (Promega, Madison, WI, USA) to produce wild-type luciferase reporter vectors (MALAT1-wt, HIF3A-wt) or mutant luciferase reporter vectors (MALAT1-mut, HIF3A-mut), respectively. The above vectors and miR-NC/miR-769-5p mimics were transfected into PDLCs by Lipofectamine 3000 (Invitrogen). After transfection of 48 h, a dual-luciferase reporter assay kit (Promega) was used to detect the luciferase activities.

### 2.10. Statistical Analysis

All data were evaluated by applying SPSS 22.0 software (IBM Corp., Armonk, NY, USA) and presented as the mean ± standard deviation. The comparisons between two groups or among multiple groups were performed by Student's *t*-test or one-way ANOVA followed by Tukey's post hoc test. A value of *P* < 0.05 was deemed as a significant difference.

## 3. Results

### 3.1. Inhibition of MALAT1 Attenuates LPS-Induced PDLC Injury

**Figure 1:**
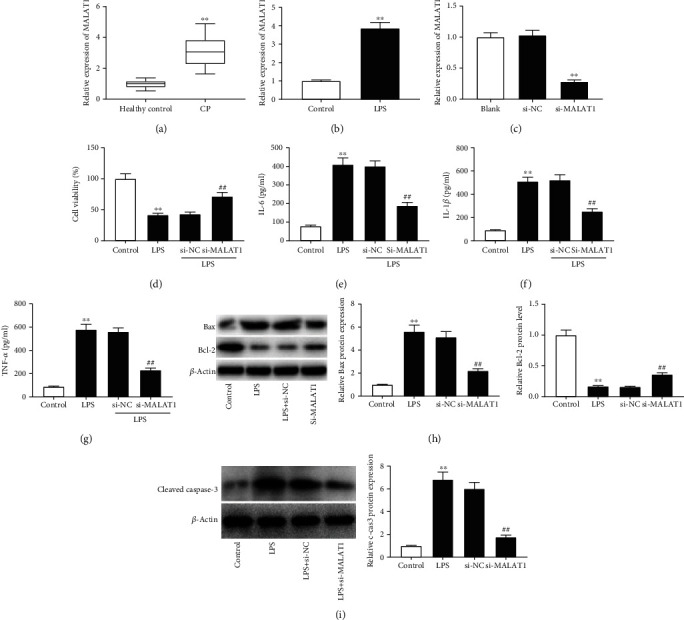
Inhibition of metastasis-associated lung adenocarcinoma transcript 1 (MALAT1) attenuated lipopolysaccharide- (LPS-) induced periodontal ligament cell (PDLC) injury. (a) The expression of MALAT1 was detected by quantitative real-time polymerase chain reaction (qRT-PCR) in gingival tissues of patients with chronic periodontitis (CP) and healthy controls. ^∗∗^*P* < 0.01 vs. healthy controls. (b) The expression of MALAT1 was detected by qRT-PCR in LPS-treated PDLCs and control cells. ^∗∗^*P* < 0.01 vs. control cells. (c) The expression of MALAT1 was detected by qRT-PCR in PDLCs transfected with si-NC or si-MALAT1. ^∗∗^*P* < 0.01 vs. si-NC. (d) Cell viability was detected by MTT assay in LPS-treated PDLCs transfected with si-NC or si-MALAT1. ^∗∗^*P* < 0.01 vs. control; ^##^*P* < 0.01 vs. si-NC. (e–g) The concentrations of interleukin- (IL-) 6, IL-1*β*, and tumor necrosis factor-*α* (TNF-*α*) were measured by enzyme-linked immunosorbent assay (ELISA) in supernatants of LPS-treated PDLCs transfected with si-NC or si-MALAT1. ^∗∗^*P* < 0.01 vs. control; ^##^*P* < 0.01 vs. si-NC. (h) The protein levels of Bax and Bcl-2 were detected by western blot in LPS-treated PDLCs transfected with si-NC or si-MALAT1. ^∗∗^*P* < 0.01 vs. control; ^##^*P* < 0.01 vs. si-NC. (i) The protein level of caspase-3 was detected by western blot in LPS-treated PDLCs transfected with si-NC or si-MALAT1. ^∗∗^*P* < 0.01 vs. control; ^##^*P* < 0.01 vs. si-NC.

MALAT1 expression was increased in gingival tissues of patients with CP compared with gingival tissues of healthy controls (*P* < 0.01, Figure 1(a)). As illustrated in Figure 1(b), MALAT1 expression in LPS-treated PDLCs was higher than that in control PDLCs (*P* < 0.01). To explore the role of MALAT1 in CP, PDLCs were transfected with si-NC or si-MALAT1. The results displayed that MALAT1 knockdown suppressed MALAT1 expression in PDLCs (*P* < 0.01, Figure 1(c)). MTT assay indicated that cell viability was inhibited in LPS-treated PDLCs compared with control PDLCs, while silencing of MALAT1 enhanced cell viability in LPS-treated PDLCs (*P* < 0.01, Figure 1(d)). Moreover, the levels of IL-6, IL-1*β*, and TNF-*α* were obviously increased in LPS-treated PDLCs compared with control PDLCs, while knockdown of MALAT1 inhibited the release of inflammatory cytokines in LPS-treated PDLCs (*P* < 0.01, Figures 1(e)–1(g)). Further studies indicated that the protein levels of Bax and caspase-3 were enhanced, whereas Bcl-2 expression was reduced in LPS-treated PDLCs compared with control PDLCs (*P* < 0.01, Figures 1(h) and 1(i)). Silencing of MALAT1 reduced the Bax and caspase-3 protein levels and enhanced Bcl-2 expression in LPS-treated PDLCs (*P* < 0.01, Figures 1(h) and 1(i)). These data revealed that silencing of MALAT1 enhanced cell viability and inhibited inflammation and apoptosis in LPS-treated PDLCs.

### 3.2. miR-769-5p Is a Target of MALAT1

**Figure 2:**
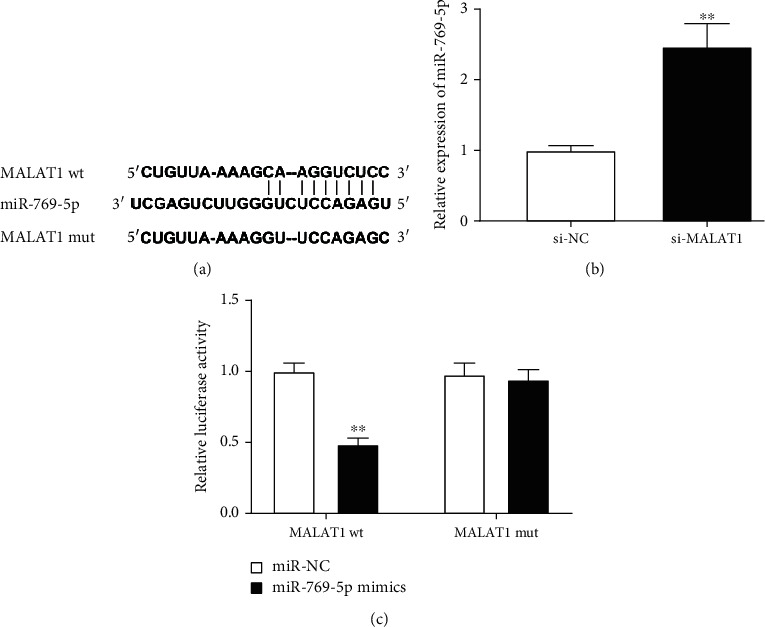
miR-769-5p was a target of MALAT1. (a) The target sites between MALAT1 and miR-769-5p were predicted by StarBase. (b) The expression of miR-769-5p was measured by qRT-PCR in PDLCs transfected with si-NC or si-MALAT1. ^∗∗^*P* < 0.01 vs. si-NC. (c) Dual-luciferase reporter (DLR) assay confirmed the relationship between MALAT1 and miR-769-5p in PDLCs. ^∗∗^*P* < 0.01 vs. miR-NC.

The underlying target site between MALAT1 and miR-769-5p was predicted using StarBase software (Figure 2(a)). To investigate the relationship between MALAT1 and miR-769-5p, si-NC or si-MALAT1 was transfected into PDLCs. The results uncovered that miR-769-5p expression in the si-MALAT1 group was increased in comparison with that in the si-NC group (*P* < 0.01, Figure 2(b)). Moreover, DLR assay showed that the luciferase activity of MALAT1-wt in the miR-769-5p mimics group was decreased as compared to that in the miR-NC group (*P* < 0.01, Figure 2(c)). These results suggested that miR-769-5p was a downstream target of MALAT1.

### 3.3. miR-769-5p Overexpression Alleviates LPS-Induced PDLC Injury

**Figure 3:**
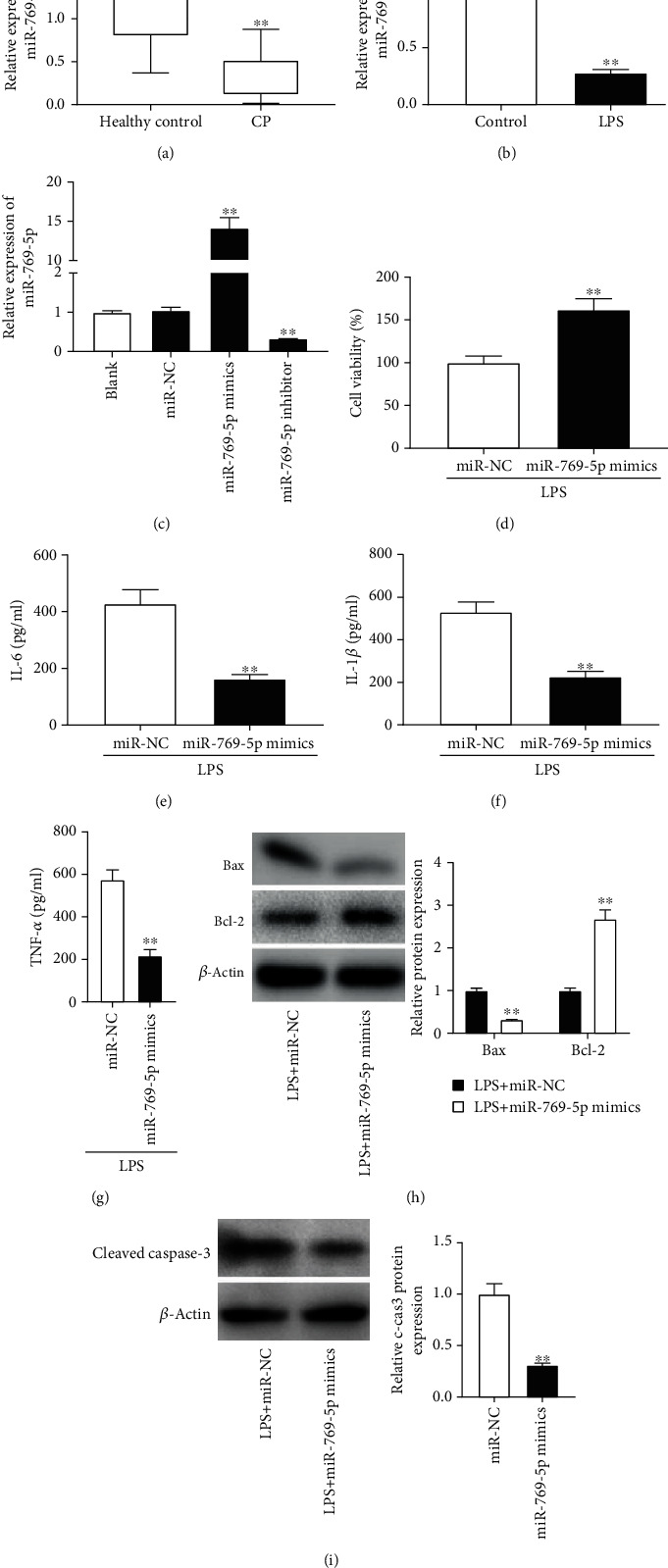
miR-769-5p mimics alleviated LPS-induced PDLC injury. (a) The expression of miR-769-5p was detected by qRT-PCR in gingival tissues of patients with CP and healthy controls. ^∗∗^*P* < 0.01 vs. healthy controls. (b) The expression of miR-769-5p was detected by qRT-PCR in LPS-treated PDLCs and control cells. ^∗∗^*P* < 0.01 vs. control cells. (c) The expression of miR-769-5p was detected by qRT-PCR in PDLCs transfected with miR-NC, miR-769-5p mimics, or miR-769-5p inhibitor. ^∗∗^*P* < 0.01 vs. the miR-NC group. (d) Cell viability was detected by MTT assay in LPS-treated PDLCs transfected with miR-NC or miR-769-5p mimics. ^∗∗^*P* < 0.01 vs. the miR-NC group. (e–g) The concentrations of IL-6, IL-1*β*, and TNF-*α* were measured by ELISA in supernatants of LPS-treated PDLCs transfected with miR-NC or miR-769-5p mimics. ^∗∗^*P* < 0.01 vs. the miR-NC group. (h) The protein levels of Bax and Bcl-2 were detected by western blot in LPS-treated PDLCs transfected with miR-NC or miR-769-5p mimics. ^∗∗^*P* < 0.01 vs. the miR-NC group. (i) The protein level of caspase-3 was detected by western blot in LPS-treated PDLCs transfected with miR-NC or miR-769-5p mimics. ^∗∗^*P* < 0.01 vs. the miR-NC group.

miR-769-5p expression was reduced in gingival tissues of patients with CP compared with gingival tissues of healthy controls (*P* < 0.01, Figure 3(a)). At the same time, miR-769-5p expression was decreased in LPS-treated PDLCs as compared to control PDLCs (*P* < 0.01, Figure 3(b)). To explore the role of miR-769-5p in CP, miR-NC, miR-769-5p mimics, or miR-769-5p inhibitor was transfected into PDLCs. We discovered that miR-769-5p mimics enhanced miR-769-5p expression in PDLCs, while miR-769-5p inhibitor caused a reverse effect (*P* < 0.01, Figure 3(c)). MTT assay demonstrated that the cell viability was enhanced in the miR-769-5p mimics group compared to the miR-NC group (*P* < 0.01, Figure 3(d)). The levels of IL-6, IL-1*β*, and TNF-*α* were obviously decreased in the miR-769-5p mimics group as compared to the miR-NC group in LPS-treated PDLCs (*P* < 0.01, Figures 3(e)–3(g)). Western blot assay showed that miR-769-5p mimics reduced the protein levels of Bax and caspase-3 and enhanced Bcl-2 expression in LPS-treated PDLCs (*P* < 0.01, Figures 3(h) and 3(i)). These data uncovered that overexpression of miR-769-5p enhanced cell viability and inhibited inflammation and apoptosis in LPS-treated PDLCs.

### 3.4. HIF3A Is a Target of miR-769-5p

**Figure 4:**
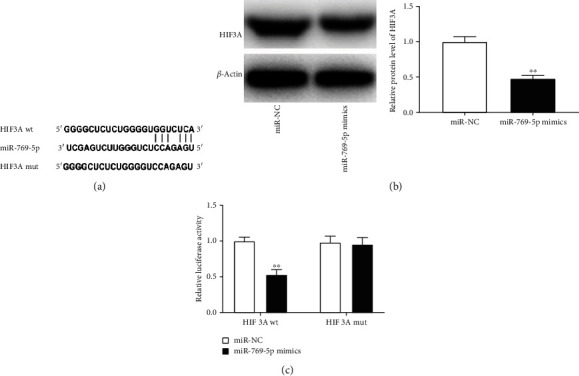
HIF3A was a target of miR-769-5p. (a) The target sites between miR-769-5p and HIF3A were predicted by StarBase. (b) The expression of HIF3A was detected by western blot in PDLCs transfected with miR-NC or miR-769-5p mimics. ^∗∗^*P* < 0.01 vs. miR-NC. (c) DLR assay confirmed the relationship between miR-769-5p and HIF3A in PDLCs. ^∗∗^*P* < 0.01 vs. miR-NC.

The potential target site between miR-769-5p and HIF3A was predicted by StarBase and TargetScan software (Figure 4(a)). To explore the relationship between miR-769-5p and HIF3A, miR-NC or miR-769-5p mimics was transfected into PDLCs. We found that the expression of HIF3A was obviously decreased in the miR-769-5p mimics group compared with the miR-NC group (*P* < 0.01, Figure 4(b)). Moreover, DLR assay displayed that miR-769-5p mimics inhibited the luciferase activity of HIF3A-wt in PDLCs (*P* < 0.01, Figure 4(c)). The above results implied that HIF3A was a direct target gene of miR-769-5p.

### 3.5. MALAT1 Knockdown Inhibits LPS-Induced PDLC Injury through Regulating miR-769-5p/HIF3A Axis

**Figure 5:**
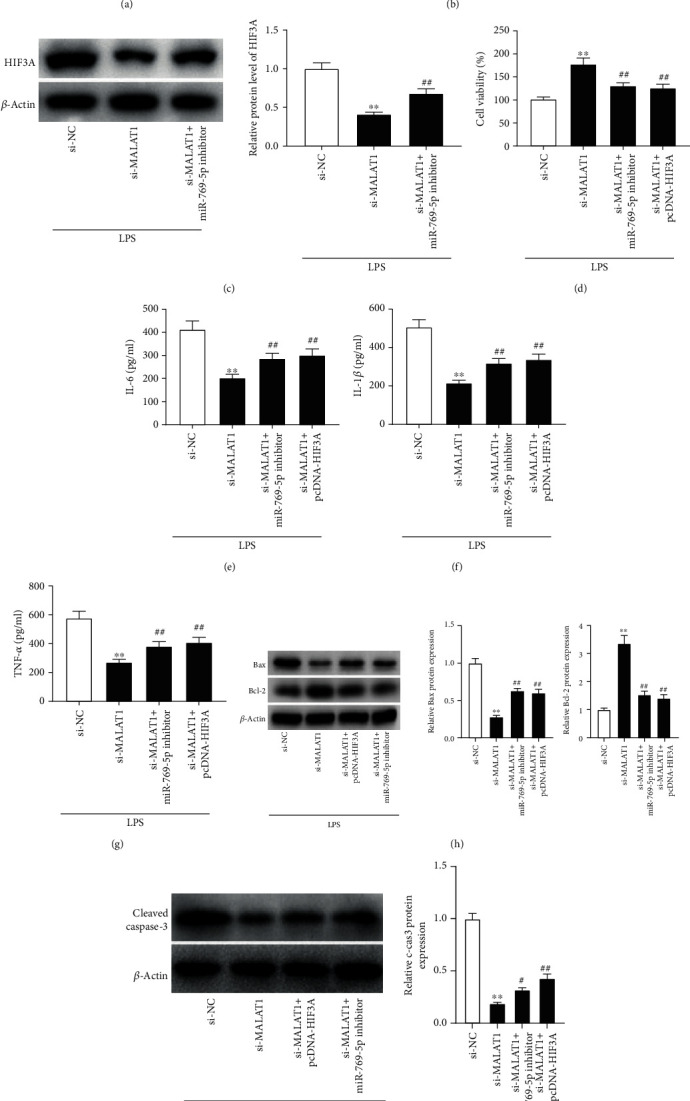
MALAT1 knockdown inhibited LPS-induced PDLC injury through regulating the miR-769-5p/HIF3A axis. (a) The expression of HIF3A was detected by qRT-PCR in gingival tissues of patients with CP and healthy controls. ^∗∗^*P* < 0.01 vs. healthy controls. (b) The expression of HIF3A was detected by western blot in LPS-treated PDLCs and control cells. ^∗∗^*P* < 0.01 vs. control cells. (c) The expression of HIF3A was detected by western blot in LPS-treated PDLCs transfected with si-NC, si-MALAT1, or si-MALAT1+miR-769-5p inhibitor. ^∗∗^*P* < 0.01 vs. si-NC; ^##^*P* < 0.01 vs. si-MALAT1. (d) Cell viability was detected by MTT assay in LPS-treated PDLCs transfected with si-NC, si-MALAT1, miR-769-5p inhibitor, or pcDNA-HIF3A. ^∗∗^*P* < 0.01 vs. si-NC; ^##^*P* < 0.01 vs. si-MALAT1. (e–g) The concentrations of IL-6, IL-1*β*, and TNF-*α* were measured by ELISA in supernatants of LPS-treated PDLCs transfected with si-NC, si-MALAT1, miR-769-5p inhibitor, or pcDNA-HIF3A. ^∗∗^*P* < 0.01 vs. si-NC; ^##^*P* < 0.01 vs. si-MALAT1. (h) The protein levels of Bax and Bcl-2 were detected by western blot in LPS-treated PDLCs transfected with si-NC, si-MALAT1, miR-769-5p inhibitor, or pcDNA-HIF3A. ^∗∗^*P* < 0.01 vs. si-NC; ^##^*P* < 0.01 vs. si-MALAT1. (i) The protein level of caspase-3 was detected by western blot in LPS-treated PDLCs transfected with si-NC, si-MALAT1, miR-769-5p inhibitor, or pcDNA-HIF3A. ^∗∗^*P* < 0.01 vs. si-NC; ^#^*P* < 0.05 and ^##^*P* < 0.01 vs. si-MALAT1.

HIF3A expression was increased in gingival tissues of patients with CP as compared to gingival tissues of healthy control (*P* < 0.01, Figure 5(a)). The relative protein level of HIF3A was increased in LPS-treated PDLCs compared with control PDLCs (*P* < 0.01, Figure 5(b)). Moreover, MALAT1 knockdown inhibited HIF3A expression in LPS-treated PDLCs, while miR-769-5p inhibition reversed this inhibitory effect (*P* < 0.01, Figure 5(c)). Then, the joint effects of silencing of MALAT1 and miR-769-5p knockdown or HIF3A overexpression on LPS-induced PDLC injury were studied. We discovered that miR-769-5p downregulation or HIF3A overexpression partly inhibited the promoting effect of MALAT1 silencing on viability of LPS-treated PDLCs (*P* < 0.01, Figure 5(d)). Besides, both miR-769-5p inhibition and HIF3A overexpression reversed the inhibitory effects of si-MALAT1 on the secretion of IL-6, IL-1*β*, and TNF-*α* in LPS-treated PDLCs (*P* < 0.01, Figures 5(e)–5(g)). Further studies showed that both the low expression of miR-769-5p and high expression of HIF3A reversed the inhibiting effects of MALAT1 knockdown on the levels of Bax and caspase-3 and the promoting effect on Bcl-2 protein level (*P* < 0.01, Figures 5(h) and 5(i)). The results suggested that MALAT1 knockdown inhibited LPS-induced PDLC injury through regulating miR-769-5p and HIF3A expression.

## 4. Discussion

CP not only seriously affects oral health but also increases the patient's risk of other chronic diseases [30, 31]. Previous researches have pointed that some lncRNAs are associated with pathogenesis of periodontitis, such as lncRNA OIP5-AS1 and LINC00687 [32, 33]. In our study, the results revealed that MALAT1 expression was enhanced in gingival tissues of patients with CP and LPS-treated PDLCs. Knockdown of MALAT1 promoted cell viability and inhibited inflammation and apoptosis in LPS-treated PDLCs. Besides, MALAT1 targeted miR-769-5p and miR-769-5p targeted HIF3A. A further study demonstrated that knockdown of MALAT1 alleviated LPS-induced PDLC injury by regulating the miR-769-5p/HIF3A axis.

Increasing studies showed that MALAT1 plays a pivotal role in LPS-induced inflammation models, such as acute lung injury [34] and ATDC5 cell inflammatory injury [35]. Recently, studies have demonstrated that MALAT1 participates in the development of periodontitis and is upregulated in PDLSCs [12] and inflammatory gingival tissues of CP [13]. In this study, MALAT1 expression was notably increased in gingival tissues of patients with CP and LPS-treated PDLCs, suggesting that MALAT1 was related to pathogenesis of CP. Previous studies demonstrated that MALAT1 overexpression promotes inflammatory cytokine production in LPS-treated HGF cells [13]. Similarly, our results suggested that knockdown of MALAT1 inhibited the secretion of inflammatory cytokines in LPS-treated PDLCs. Meanwhile, we found that MALAT1 knockdown enhanced cell viability and inhibited cell apoptosis in LPS-treated PDLCs. All these results suggested that MALAT1 knockdown may inhibit the occurrence and developments of CP in vitro.

Previous studies showed that MALAT1 is involved in CP pathogenesis by sponging miR-125a-3p [11] or miR-20a [13]. In this study, we found that miR-769-5p was a downstream target of MALAT1 and reversely modulated by MALAT1. Numerous studies indicated that miR-769-5p participates in the growth of some cancers and is reduced in non-small-cell lung carcinoma (NSCLC) [36] and retinoblastoma (RB) [37]. Interestingly, Du et al. have pointed that miR-769-5p expression is decreased in LPS-treated PDLCs [19]. Similarly, our results displayed that miR-769-5p expression was reduced in LPS-treated PDLCs and gingival tissues of patients with CP, suggesting that miR-769-5p may be an anti-inflammatory gene in CP. At present, the function of miR-769-5p has been explored in several types of human cancers. For example, miR-769-5p silencing can inhibit cell viability in gastric cancer cells [38]. Silencing of miR-769-5p obviously inhibits cell viability and promotes cell apoptosis in glioma cell lines [39]. However, the function of miR-769-5p is rarely discussed in inflammatory diseases. In this study, our results cleared that miR-769-5p overexpression enhanced cell viability and inhibited apoptosis in LPS-treated PDLCs. Besides, our study revealed that miR-769-5p overexpression could inhibit the secretion of inflammatory cytokines in LPS-treated PDLCs. The above results suggested that miR-769-5p makes a great deal of contributions on inhibiting the development of CP in vitro. Because MALAT1 directly targeted miR-769-5p, we speculated that MALAT1 knockdown may suppress CP progression by targeting miR-769-5p.

HIF3A, a main gene involved in the homeostatic processes, is commonly involved in chronic inflammation [26]. As a transcription factor for many target genes, HIF3A can be regulated by miR-210 in LPS-treated PDLCs [40] or modulated by miR-429 in human endothelial cells [41]. In this study, HIF3A was a target gene of and negatively regulated by miR-769-5p. Previous researches revealed that HIF3A expression is increased in periodontitis [40] and in LPS-treated BV-2 microglial cells [42]. Similar to previous results, we found that HIF3A expression was also increased in gingival tissues of patients with CP and LPS-treated PDLCs, suggesting that HIF3A may be a proinflammatory gene in CP development. In addition, Cuomo et al. have discovered that HIF3A is involved in inflammatory cell infiltration in a murine model of arteriotomy [43]. Meanwhile, HIF3A can regulate the inhibition effect of miR-210 on secretion of inflammatory factors and cell apoptosis in LPS-treated PDLCs [40]. Thus, we speculated that miR-769-5p overexpression may inhibit inflammation and apoptosis by regulating HIF3A in LPS-treated PDLCs. Further studies revealed that miR-769-5p inhibition and HIF3A overexpression reversed the influence of MALAT1 silencing on cell viability, inflammatory factor secretion, and apoptosis-related protein levels in LPS-treated PDLCs. Because MALAT1 negatively regulated miR-769-5p and miR-769-5p negatively regulated HIF3A, we speculated that MALAT1 knockdown may alleviate LPS-induced PDLC injury by regulating the miR-769-5p/HIF3A axis.

## 5. Conclusion

In conclusion, this research revealed that the expression of MALAT1 was upregulated in gingival tissues of patients with CP and LPS-treated PDLCs. In addition, knockdown of MALAT1 enhanced cell viability and inhibited inflammation and apoptosis in LPS-treated PDLCs through regulating the miR-769-5p/HIF3A axis. This study may provide a new target for the therapy of CP.

## Figures and Tables

**Table 1:** Primers for quantitative real-time polymerase chain reaction (qRT-PCR).

Gene	Sequences (5′-3′)
MALAT1-F	AAAGCAAGGTCTCCCCACAAG
MALAT1-R	GGTCTGTGCTAGATCAAAAGGCA
miR-769-5p-F	ACACTCCAGCTGGGTGAGACCTCTGGGTTCTG
miR-769-5p-R	CTCAACTGGTGTCGTGGA
HIF3A-F	CTTTCTGCTCTTTCCTCTCAGC
HIF3A-R	GCTCATTCAGGTTCAGGAGTG
GAPDH-F	GCGAGATCGCACTCATCATCT
GAPDH-R	TCAGTGGTGGACCTGACC
U6-F	CTCGCTTCGGCAGCACA
U6-R	AACGCTTCACGAATTTGCGT

## Data Availability

All data can be obtained by contacting the corresponding author.
